# An In Vivo Study to Compare the Clinical Effectiveness of Clear Retainer Made on a Conventional and a Digitally Fabricated Model Over a Six-Month Period After Debonding

**DOI:** 10.7759/cureus.54740

**Published:** 2024-02-23

**Authors:** Perthish Sharma, Sukhdeep Singh Kahlon, Chetan Dev Singh Boparai

**Affiliations:** 1 Orthodontics and Dentofacial Orthopedics, Sri Guru Ram Das Institute of Dental Sciences and Research, Amritsar, IND

**Keywords:** digital orthodontics, orthodontic appliances, debonding, retention, essix retainer

## Abstract

Background

With the advent of 3D printing, many more possibilities have arisen for treatment planning. 3D rapid prototyping has enabled us to see a whole other dimension that has helped us to give the best possible care for our patients. With more and more advancements being made in this subject, it becomes necessary to check the reliability of the equipment and its effectiveness in the management of the problem at hand. This original study was conducted with the aim of checking the accuracy, dimensional stability, and reliability of orthodontic retainers made on a conventional and digitally fabricated model over a six-month period after debonding.

Material and methods

The patients were selected from those who have completed fixed orthodontic mechanotherapy from the Department of Orthodontics and Dentofacial Orthopaedics, Sri Guru Ram Das Institute of Dental Sciences and Research, Sri Amritsar. Fifty patients received a clear retainer, which was fabricated for the upper and lower arch after removing the brackets. Patients were included in this study irrespective of their age groups. The manual method used a vacuum-forming machine to fabricate six retainers on stone models. In the digital method, new impressions were taken after three months, and digital models were obtained through 3D scanning and printing, followed by clear retainer fabrication. The data were gathered through a systematic process involving manual and digital methods for clear retainer fabrication and subsequent evaluation. The data obtained was computed for statistical evaluation and comparison.

Results

Mean and standard deviations of conventional (manual) and digital variables in the two groups were calculated. An ANOVA test was used to evaluate statistically significant differences for mesiodistal width and buccolingual width, and a post hoc Tuckey test was applied for multiple comparisons. The results indicated that most mesiodistal and buccolingual width measurements showed non-significant variations and exhibited a good correlation. Extraction space opening, assessed through an independent t-test for both the maxilla and mandible, also yielded non-significant and comparable results. Additionally, intra-operator and inter-operator measurements using a digital caliper demonstrated high agreement. Intra-class correlation (ICC) values exceeded 0.75, and inter-operator ICC results reflected a high level of agreement ranging from 0.8 to 0.99.

Conclusion

The primary objective of this study was to establish a correlation between the accuracy, dependability, and clinical efficacy of orthodontic retainers produced using both conventional and digitally created models. This investigation spanned a duration of six months following the removal of orthodontic brackets. The results showed that most of the statistically significant values were due to the inherent potential of the 3D printer for polymerization shrinkage, which, being a stereolithographic 3D printer, had a potential for a slight dimensional shift in the transverse dimension. However, the mean difference between all the models printed was slight and clinically insignificant.

## Introduction

The utilization of computer-aided design and manufacturing (CAD/CAM) technology for the fabrication of orthodontic retainers has been thoroughly examined. Literature has investigated the precision and dependability of measurements derived from digital models in contrast to those obtained through conventional models [1-3]. CAD/CAM devices play a crucial role in post-orthodontic treatment, reducing the risk of relapse and aiding in maintaining tooth alignment. Several factors contribute to relapse, such as periodontal impact, unstable tooth positioning, and ongoing skeletal growth [4]. This prompts the use of retainers to prevent teeth from reverting until gingival and periodontal restructuring, along with skeletal growth, reach a significant stage of completion.

Retainers are custom-designed orthodontic devices that preserve and stabilize the alignment of teeth, facilitating the reorganization of surrounding tissues. Removable retainers, a common retention method, offer various options, with the Hawley retainer (HR) and vacuum-formed retainer (VFR) being the most prevalent. The HR, a century-old appliance created by Charles Hawley in 1919, remains a prominent choice [5]. In contrast, the VFR, also known as the clear retainer, introduced in 1971, boasts advantages such as transparency, ease of production, quick insertion, enhanced aesthetics, and fewer adjustments [6]. Despite its benefits, VFRs may experience occlusal wear and decreased vertical settling over time [7]. Nonetheless, their cost-effectiveness and preference over Hawley's retainers in various disciplines due to reduced social discomfort and lower susceptibility to damage make them a popular choice [8].

This study aims to clinically evaluate the efficacy of clear retainers produced through both conventional and digitally manufactured models over a six-month period. A six-month period strikes a balance between obtaining meaningful data and reducing the burden on both researchers and participants, considering study logistics and patient compliance.

## Materials and methods

The present study evaluated the accuracy, reliability, and outcome of an orthodontic removable clear retainer fabricated using conventional models made from alginate impression material and another clear retainer fabricated on a digitally fabricated cast of the same patient. A conducted statistical power analysis indicated that a sample size of 50 possesses adequate statistical power to discern meaningful differences between the two groups in the study comparing clear retainers on conventional and digitally fabricated models over a six-month period.

Fifty subjects, regardless of age, who completed fixed orthodontic treatment were included. Permission to carry out the study was obtained from the Institutional Ethical Committee of Baba Farid University of Health Sciences (BFUHS/2K19/P-TH/13217). Inclusion criteria focused on clinically acceptable tooth alignment, facial symmetry, and patients at the debonding stage ready for retainer placement. Exclusion criteria covered specific conditions such as torus, cleft lip, spacing, crowding, rotations, asymmetry, periodontal disease history, congenital abnormalities, and prosthetic needs post-treatment.

Materials included perforated impression trays, alginate impression material, bowl, mixing spatula, class III stone for models, orthodontic base formers, digital vernier calipers, three-shape version E2 digital desktop scanner (3 Shape A/S, Copenhagen, Denmark), FormLabs, Form 2 3D Printer (FormLabs Inc, Somerville, US) and Scheu MiniStar vacuum (Scheu-Dental GmbH, Iserlohn, Germany) forming machine.

In the manual method, the selected patients who had completed their orthodontic treatment were given a set of retainers fabricated on a model made from type III stone material. After the retrieval of the stone models, they were used to construct a clear retainer on the maxillary as well as the mandibular set. Each set of vacuum form retainers was constructed by the same person using a Scheu Minister vacuum forming machine (version 2017) using standard instructions provided by the manufacturer. The thermoform material used for the fabrication of the clear retainer was Duran plus 1 mm thick sheet (Scheu dental pressure molding material; Scheu-Dental GmbH, Iserlohn, Germany), and the pressure and temperature at which the retainer was fabricated were according to manufacturer recommendation (temperature of 220° C and 4 bar pressure). The foil was subsequently adjusted to achieve a 1-2 mm extension in the gingival region, as well as in the palatal/lingual region and for occlusal coverage of the second molar. This was accomplished using a laboratory handpiece and a range of burs.

**Figure 1:**
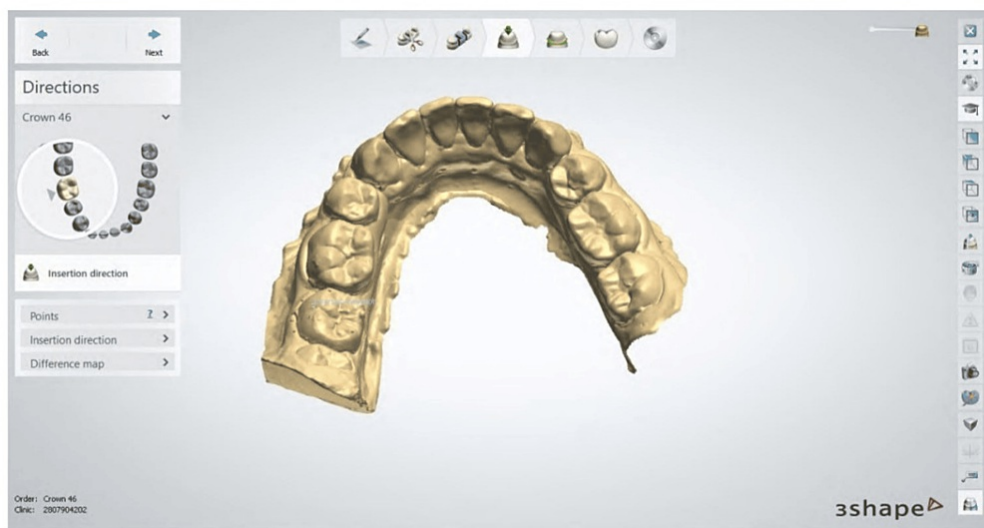
Scanning of digital models for conversion into stl file

In the digital method, after a span of three months, the same patient was recalled, a fresh set of impressions was taken using the same methodology as the manual method, and the new models obtained were scanned using the 3D laser scanner (version E2) to produce a 3D scan of the same model in a binary stereolithographic (stl) format as shown in Figure 1. The digital models underwent editing utilizing the exclusive PreForm® 3D printing software developed by Formlabs, Somerville, US. During the editing process, the study models were refined by trimming away non-essential areas, specifically focusing on retaining only the necessary working components. This approach aimed to streamline the 3D printing process and minimize associated manufacturing expenses without altering the overall scale of the models.

**Figure 2:**
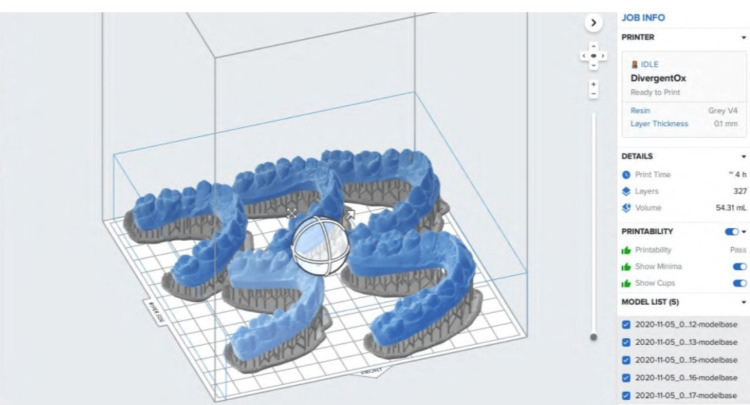
Preparing models to print using Formlabs printer (Formlabs Inc., Somerville, US)

After trimming the digital model as desired, it was prepared for digital printing using a Formlabs version 2 digital printer. All the 3D models were then prepared for nesting using the same software, with adjustments made according to the base of the printing plate, as depicted in Figure 2. In a single flow, around six models were printed using the printer to save time and material. These printed models were then processed after the completion of the cycle and were made ready for the fabrication of the clear retainer using the same criteria as the manual method.

**Figure 3:**
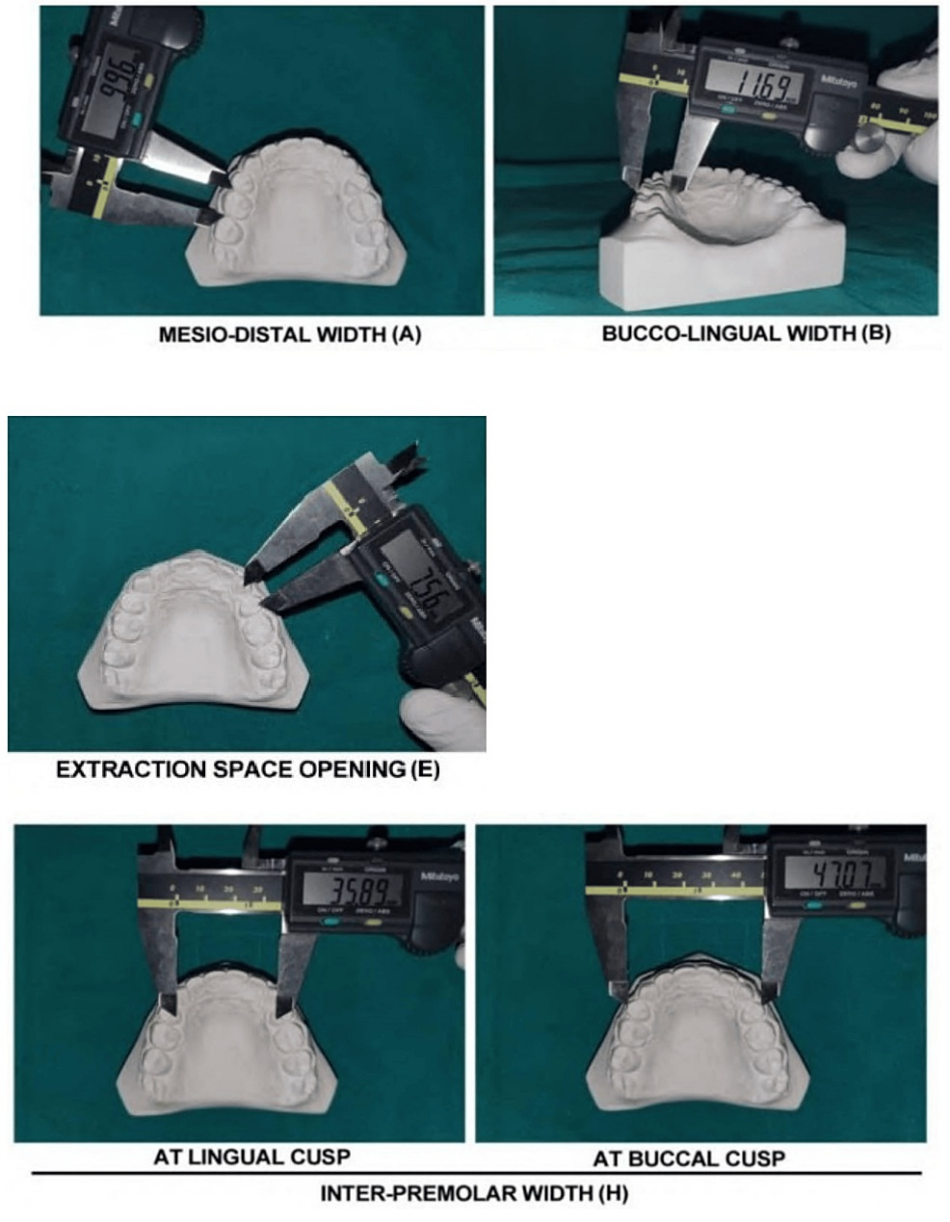
Measurements of manually fabricated retainer

**Figure 4:**
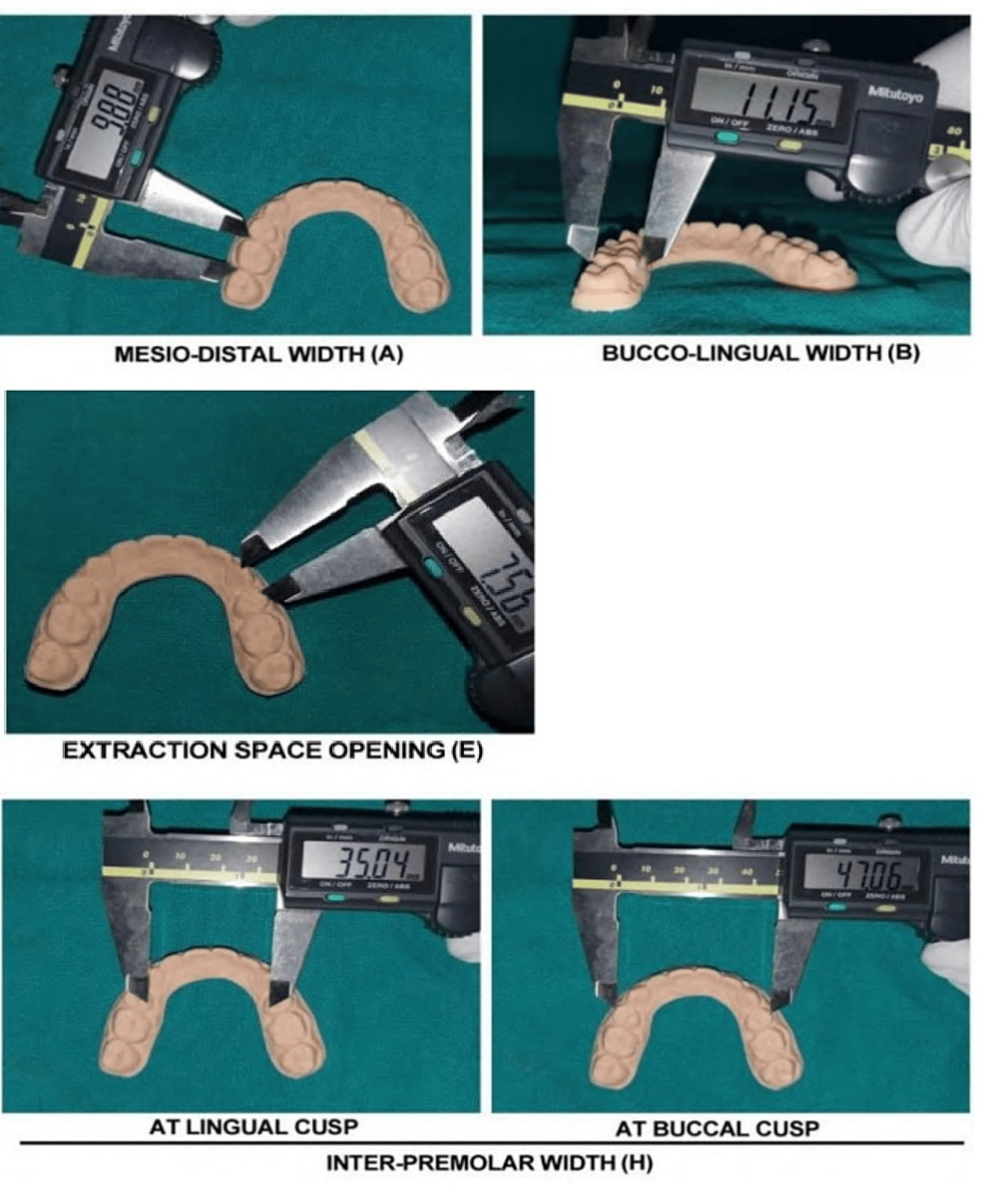
Measurements of digitally fabricated retainer

In order to evaluate the clinical acceptability of the models, measurements (at baseline and at six months) were conducted on parameters that were deemed clinically important. These parameters are outlined as follows: (1) inter-premolar width (spatial separation between the buccal cusp tips and the lingual cusp tip of premolars); (2) extraction space opening (the sum of contact point displacements in the anteroposterior plane where extractions were carried out); (3) buccolingual width (the maximum breadth, measured perpendicular to the mesiodistal dimension, between the buccal and lingual surfaces); (4) mesiodistal width measurement (the measurement of the distance between the mesial and distal points of contact is conducted using calipers that are positioned perpendicular to the occlusal surfaces). Figures 3-4 demonstrate the measurements in manual and digital methods, respectively.

The SPSS software (version 24.0.1; IBM, Inc., Armonk, US) was employed for data analysis. Reliability values for specific parameters were assessed through the intraclass correlation coefficient (ICC), measuring both intra-examiner and inter-examiner reliability. A score below 0.4 indicated poor reliability, while a value above 0.75 was considered excellent. To address potential statistical errors from repeated analyses, parameters were evaluated by examining variations in tooth size across different planes and arch dimensions. Means and standard deviations were computed using SPSS, and accuracy between the two types of models was assessed through ANOVA, chi-square test, Student t-test, and post hoc Tukey test.

## Results

In the study sample of 50 sets of models, the general fine details of the models were incrementally reduced as the models transformed from stone to digital and then to rapid prototyping digital models. Stone models generally have a smooth surface and show well-defined boundaries of the interproximal contact points and cervical margins that demarcate the boundary of each tooth. Minor artifacts like air bubbles were observed but were considered negligible because they were small and far from the anatomical landmark. On the other hand, the digitally printed rapid-prototyped models were a bit coarser and less distinct in their anatomical representation as compared to the stone plaster models, respectively.

**Figure 5:**
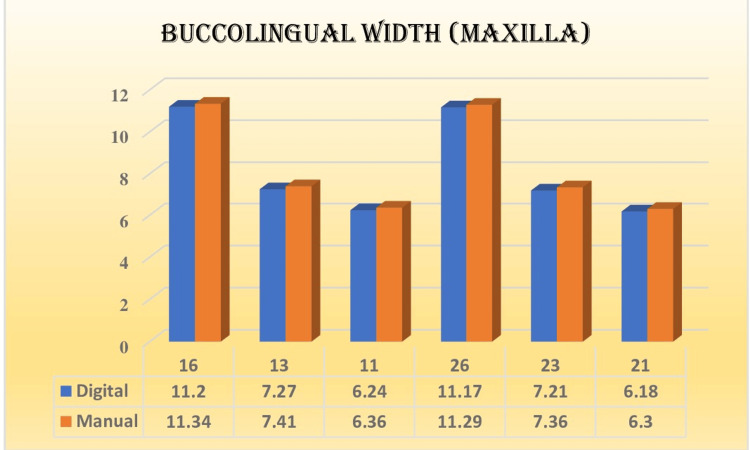
Mean values of buccolingual width (maxilla in mm) in different teeth fabricated by two methods

**Figure 6:**
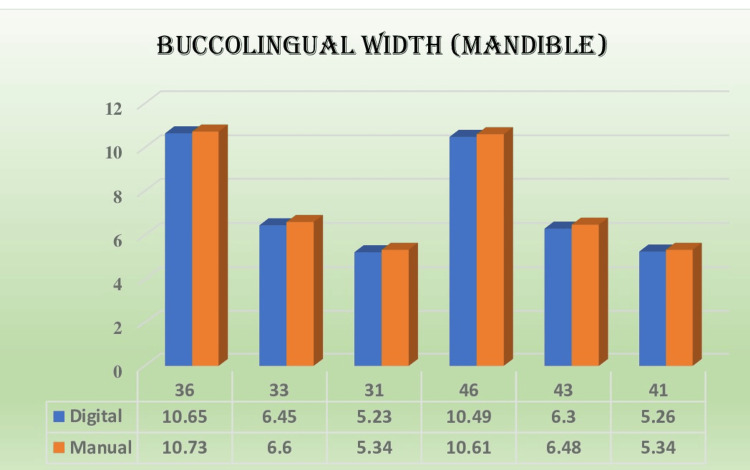
Mean values of buccolingual width (mandible in mm) in different teeth fabricated by two methods

**Figure 7:**
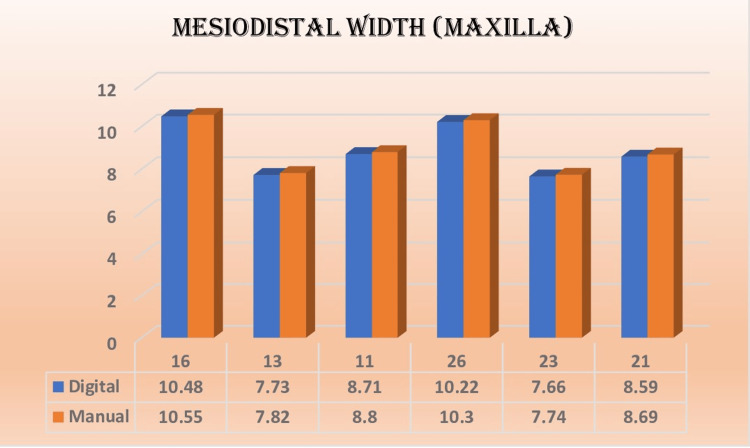
Mean values of mesiodistal width (maxilla in mm) in different teeth fabricated by two methods

**Figure 8:**
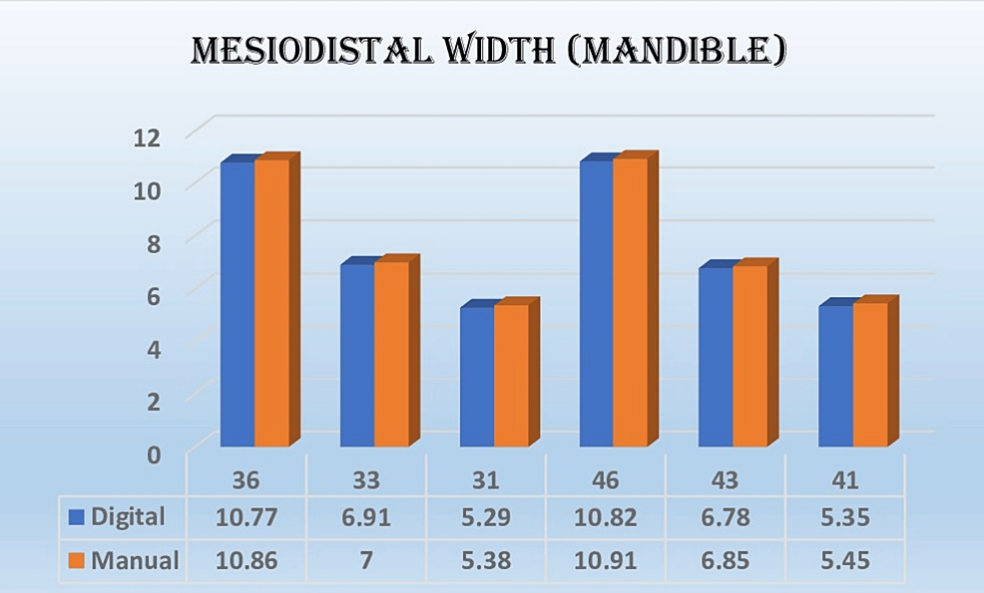
Mean values of mesiodistal width (mandible in mm) in different teeth fabricated by two methods

The results indicated that most of the measurements obtained in relation to the mesiodistal width and buccolingual width were non-significant and showed good correlation with each other, as shown in Figures 5-8. The extraction space opening was also subjected to statistical analysis with the help of an independent t-test for the maxilla and the mandible, which also showed non-significant and comparable results.

Also, as seen from the computed data tables (Tables 1-5), we can have a good estimation of the inter-premolar width, which came out to be clinically insignificant.

**Table 1:** Paired t-test for testing the significant difference between readings of digitally fabricated and manually fabricated models for maxilla NS - not significant

Location	Mean value		Mean difference	t-value	D.F.	p-value	Remark
	Digital	Manual					
Buccal cusp tip	44.164	44.224	-0.060	-1.703	49	0.09484	NS
Lingual cusp tip	34.312	34.316	-0.004	-0.031	49	0.97537	NS

**Table 2:** Paired t-test for testing the significant difference between readings of digitally fabricated and manually fabricated models for mandible NS - not significant; *** - significant

Location	Mean value		Mean difference	t-value	D.F.	p-value	Remark
	Digital	Manual					
Buccal cusp tip	37.677	37.734	-0.057	-0.724	43	0.47328	NS
Lingual cusp tip	29.548	29.691	-0.143	-3.615	43	0.00078	***

**Table 3:** Independent t-test for testing the significant difference between extraction space opening (mm) at maxilla and mandible locations NS - not significant

Location	Mean value	Mean difference	t-value	D.F.	p-value	Remark
Maxilla (n=50)	0.212	-0.024	-1.066	93	0.28927	NS
Mandible (n=45)	0.236					

**Table 4:** Tukey post-hoc paired comparison test between mean values of mesiodistal width (mandible and maxilla) of the two fabrication methods NS - not significant

Tooth no	Paired comparison	Observed difference	p-value	Remark
Mandible				
36	manual-digital	0.10	0.999978	NS
33	manual-digital	0.08	0.999994	NS
31	manual-digital	0.09	0.999983	NS
46	manual-digital	0.09	0.999987	NS
43	manual-digital	0.07	0.999999	NS
41	manual-digital	0.10	0.999956	NS
Maxilla				
16	manual-digital	0.07	0.999997	NS
13	manual-digital	0.09	0.999945	NS
11	manual-digital	0.10	0.999916	NS
26	manual-digital	0.09	0.999965	NS
23	manual-digital	0.08	0.999990	NS
21	manual-digital	0.10	0.999897	NS

**Table 5:** Tukey post-hoc paired comparison test between mean values of buccolingual width (mandible and maxilla) of the two fabrication methods NS - not significant

Tooth no	Paired comparison	Observed difference	p-value	Remark
Mandible				
36	manual-digital	0.09	0.99999	NS
33	manual-digital	0.14	0.99883	NS
31	manual-digital	0.11	0.99990	NS
46	manual-digital	0.12	0.99973	NS
43	manual-digital	0.18	0.98913	NS
41	manual-digital	0.08	0.99999	NS
Maxilla				
16	manual-digital	0.14	0.99906	NS
13	manual-digital	0.14	0.99937	NS
11	manual-digital	0.12	0.99986	NS
26	manual-digital	0.12	0.99981	NS
23	manual-digital	0.15	0.99878	NS
21	manual-digital	0.12	0.99981	NS

The measurements conducted using a digital caliper demonstrated high agreement between intra-operators, as indicated by the intra-class correlation (ICC) values over 0.75. The inter-operator ICC results demonstrated a high level of agreement, ranging from 0.8 to 0.99.

## Discussion

Advances in the product-based industry are becoming more common, and 3D printing improves the planning and treatment of orthodontics in the field. Currently, the application of 3D printing in orthodontics has mainly been limited to the fabrication of physical models of dentition, which are clinically accurate for treatment planning and appliance fabrication. Several prior studies have cited and contrasted various forms of 3D printing methodologies, such as those by Hazeveld et al., in their examination of dental models fabricated utilizing polyjet, direct light printing, and fused filament fabrication printing procedures [9]. The study revealed variations in the clinical crown height and mesiodistal widths among dental models produced using different printing methods. Specifically, the polyjet, direct light printing, and 3D printers exhibited differences of -0.02 mm, 0.04 mm, and 0.25 mm, respectively, in clinical crown height. Similarly, the mesiodistal widths displayed differences of -0.08 mm, -0.05 mm, and -0.05 mm, respectively. Both the methods stated above vary, but there was no substantial difference between direct measurements made on a plaster model and indirect measurements scanned with a 3D VECTRA® scanner, according to a report by Metzler et al. [10].

In the present study, the mesiodistal width was measured individually for tooth numbers 16, 13, 11, 21, 23, 26 for the maxillary arch and 36, 33, 31, 41, 43, and 46 for the mandibular arch, respectively. These tooth numbers were selected to observe the reproducibility of the digital 3D printer on the X-axis, Y-axis, and Z-axis. Salmi et al. documented a significant level of consistency in the measurements obtained from a model, which were evaluated using a reference point measuring 10 mm [11]. The mesiodistal width measurements showed a lower susceptibility to error due to the utilization of a measurement technique that involved assessing the distance between points perpendicular to the mesiodistal contact point of the respective tooth.

Kim et al. evaluated the accuracy and fidelity of dental models produced using various 3D printing methods [12]. Various printers utilize diverse printing techniques, including stereolithographic apparatus, digital light processing, fused filament fabrication, and polyjet. The researchers concluded that there were notable distinctions in precision among the polyjet and digital light printing methods, as well as in the accuracy of tooth trueness. These findings contrasted the fused filament manufacturing and stereolithographic apparatus techniques. For the buccolingual width, the selection of teeth was like that of the previously mentioned mesiodistal criteria. The overall mean for the buccolingual width for the digital method was found to be 8.21 mm and 8.34 mm for the manual method in the case of the maxilla and 7.40 mm and 7.52 mm for the mandible, respectively. As for the ANOVA, the results were 898.349 for the maxilla and 1087.568 for the mandible, which were statistically significant. However, compared with Tuckey's post hoc analysis, the mean of the digital and manual methods used at the individual tooth level found no significant difference in the measurements regarding the buccolingual width of the maxilla and mandible, respectively.

According to a study by Fleming et al., a comparison was made between digitally printed and conventional models, which stated the evidence for the validity of the digital models and concluded that the observed difference between the digital and conventional models was likely to be clinically acceptable and could be used for orthodontic purposes [13]. Keating et al. examined one stereolithographic replica and concluded that high-built layer thickness would adversely affect the printed model in the x-plane, y-plane, and z-plane [14]. In the stereolithographic printing technique, models were printed in a horseshoe-shaped configuration to help print only the desired area of interest. However, this could also have affected the transverse dimension of the models, as supported in the literature [15]. Tahir et al. conducted a crossover, randomized control trial aimed to compare vacuum-formed thermoplastic retainers (VFRs) constructed on stone models (VFR-CV) and 3D printed models (VFR-3D) concerning patients' perspectives and post-treatment stability [16]. The study involved 30 subjects, with 13 in group A (VFR-CV first) and 14 in group B (VFR-3D first). Little's Irregularity Index (LII) indicated an increase in group A after wearing both VFRs, while group B showed no significant increase. Both groups reported improved Oral Health-Related Quality of Life (OHRQoL) after the first intervention, with no significant differences after the second. Overall, VFRs on 3D printed models demonstrated comparable OHRQoL and stability to conventionally made retainers. The inter-premolar width was measured as the distance between the two buccal and lingual cusp tips. The paired t-test was employed to assess the significance level between the two groups. The mean values for the maxillary model at the buccal cusp tip (44.164) and the lingual cusp tip (34.312) for the digital group were nearly comparable to those of the manual group, which were 44.224 at the buccal cusp tip and 34.316 at the lingual cusp tip. The p-values were 0.0958 and 0.9754 for the buccal and lingual cusp tips, respectively, proving the nonsignificant results. In the mandibular model, however, the mean in relation to the buccal cusp tip and the lingual cusp tip for the digital model was 37.677 and 29.548, respectively, and for the manual readings, it was 37.734 and 29.691. The p-value for the buccal cusp tip was 0.4732 (nonsignificant) and 0.00078 (significant) for the lingual cusp tip.

The study by Camardella T et al. also concluded that due to photopolymerization shrinkage of the material after printing with a stereolithographic 3D printer, there was a statistical significance between the digitally printed models in the transverse dimension [17]. They used a 400-W ultraviolet curing light for their study to cure the models, which were divided into two groups, namely supported groups having a supporting beam at the premolar interface and the other group that did not have any support. They found out that with the supporting beam intact, there was less dimensional change as compared to the non-supporting group after the curing process was completed.

In relation to the maxilla, the mean value of the extraction space opening was 0.212 mm, whereas the same concerning the mandible was 0.236 mm. The difference between the two (-0.024) was tested (through an independent t-test) to be statistically nonsignificant because the p-value (0.28927) was far higher than the critical limit of 0.05. The extraction space opening was measured as the effective difference measured by subtracting the values obtained from the digital models from those of the manual models. The measurement was obtained by assessing the distance between the cusp tip of the canine tooth and the buccal cusp tip of the second premolar.

The observed discrepancy between the digital and manual readings was found to be minimal in terms of its impact. According to Meade and Millett, orthodontists frequently advise using a clear retainer sheet with thicknesses of 0.75 mm and 1 mm [18]. However, a study by Zhu et al. revealed no statistically significant distinction in terms of survival rate, failure rate, or comfort between the six retainer sheets of 0.75 mm and 1 mm [19]. Therefore, in the current study, utilizing a clear retainer sheet with a thickness of 1 mm was deemed preferable.

In terms of comparing the clear retainer to that of Hawley's, there is insufficient data to determine the best option available. However, certain studies [19] have assessed many dental measurements, including inter-canine width, arch length, and intermolar width, and have shown no statistically significant differences between the groups. Due to the aesthetic concerns and much better patient perception of the clear retainer, more preference can be given to clear retainers [20]. Rowland et al. evaluated and compared the clinical efficacy of the clear and Hawley retainers following fixed orthodontic treatment, whether extraction or non-extraction, for a period of six months [21]. Among the subjects included in the extraction group, the clear retainer was utilized for 68 individuals, whereas the Hawley retainer was employed for 66 individuals. The author found a lack of statistical significance in relation to the rotation, inter-canine, and intermolar widths between the groups. In relation to the index of minor irregularities, it was seen that the clear retainer demonstrated greater efficacy in the labial portions of both the maxillary and mandibular arches when compared to Hawley's retainer. Another study done by Gardner et al. also concluded that polyethylene-based materials (like Duran Plus) are 3.7 times more resistant to wear than compared to other polypropylene-based materials, thus giving us an idea of why clear retainers are more useful in maintaining the arch dimension with respect to rotations [22].

Limitations

The sample size of the study size may limit the generalizability of findings to a broader population. A larger and more diverse sample could provide more robust insights into the clinical effectiveness of clear retainers. The six-month duration for clinical effectiveness evaluation may not capture long-term outcomes. A more extended follow-up period would be essential to assess the stability and durability of clear retainers over an extended timeframe. Factors such as oral hygiene practices, dietary habits, and individual patient responses to retention may contribute to variability in clinical effectiveness. Controlling for these variables can be challenging and may introduce confounding effects.

## Conclusions

The advent of rapid prototyping has brought about a transformative impact on both dental and medical practices, particularly in crafting tailored appliances that cater to the unique needs of individual patients. This technological leap has significantly reduced the time required for diagnosis and treatment planning. The application of 3D printing techniques has further advanced, enabling the direct printing of custom-fabricated digital aligners for precise tooth alignment. In conclusion, the study involving 50 sets of models revealed a progressive reduction in general fine details as models transitioned from stone to digital and then to digitally printed rapid prototyped models. While stone models exhibited a smooth surface and well-defined boundaries, digitally printed models appeared somewhat coarse and less distinct in their anatomical representation.

However, the measurements related to mesiodistal and buccolingual width showed non-significant variations. It also demonstrated a good correlation, indicating comparable precision between the two manufacturing methods. The analysis of extraction space opening using independent t-tests for the maxilla and mandible also yielded non-significant and comparable results. Notably, inter-premolar width estimation was clinically insignificant, suggesting both manufacturing methods provide similar outcomes in this aspect. The use of a digital caliper demonstrated high agreement between intra-operators, as demonstrated by ICC values exceeding 0.75. Additionally, inter-operator ICC results indicated a high level of agreement, ranging from 0.8 to 0.99, affirming the precision and reproducibility of measurements regardless of the manufacturing method. These findings collectively support the effectiveness of digitally manufactured retainers in maintaining alignment after orthodontic treatment. It also signifies the comparable precision between traditional and digital model creation methods.
